# Artificial Intelligence in Clinical Health Care Applications: Viewpoint

**DOI:** 10.2196/12100

**Published:** 2019-04-05

**Authors:** Michael van Hartskamp, Sergio Consoli, Wim Verhaegh, Milan Petkovic, Anja van de Stolpe

**Affiliations:** 1 Philips Research Eindhoven Netherlands

**Keywords:** artificial intelligence, deep learning, clinical data, Bayesian modeling, medical informatics

## Abstract

The idea of artificial intelligence (AI) has a long history. It turned out, however, that reaching intelligence at human levels is more complicated than originally anticipated. Currently, we are experiencing a renewed interest in AI, fueled by an enormous increase in computing power and an even larger increase in data, in combination with improved AI technologies like deep learning. Healthcare is considered the next domain to be revolutionized by artificial intelligence. While AI approaches are excellently suited to develop certain algorithms, for biomedical applications there are specific challenges. We propose six recommendations—the 6Rs—to improve AI projects in the biomedical space, especially clinical health care, and to facilitate communication between AI scientists and medical doctors: (1) Relevant and well-defined clinical question first; (2) Right data (ie, representative and of good quality); (3) Ratio between number of patients and their variables should fit the AI method; (4) Relationship between data and *ground truth* should be as direct and causal as possible; (5) Regulatory ready; enabling validation; and (6) Right AI method.

## Introduction

The idea of artificial intelligence (AI) has a long history. Since the 1950s there have been several revolutionary promises of AI replacing human work within a few decades. It turned out, however, that reaching intelligence at human levels was more complicated, which led to several “AI winters,” where interest in AI disappeared [1]. Currently, we are experiencing a renewed interest in AI, fueled by an enormous increase in computing power and an even larger increase in data generation. In combination with improved algorithms that allow training of deep neural networks, several high-tech companies have reached successes in performing tasks that are close to human or even beyond human performance: playing games like chess and Go, image recognition and computer vision, natural language processing, machine translation, and self-driving cars are just a few examples.

Health care is considered the next domain to be revolutionized by AI [2-5]. In addition to many academic efforts, companies are also getting involved. IBM has developed Watson for several health applications, such as Watson for Oncology and Watson for Genomics, and there is a large number of start-ups addressing all possible aspects of the health continuum [6,7].

## Artificial Intelligence

The term *artificial intelligence* is used to indicate development of algorithms that should execute tasks that are typically performed by human beings and are, therefore, associated with intelligent behavior. AI makes use of a variety of techniques, such as deep learning, but also probabilistic methods like Bayesian modeling [8,9]; for definitions, see He et al [2]. Colloquially, the term is applied to a machine that mimics cognitive functions, such as learning and problem solving [2,4,10,11].

## Use of Artificial Intelligence Methods in Health Care

It is clear that health care has numerous needs that could benefit from solutions developed with, or by embedding, artificial intelligence [2,4,8]. In this brief article, we focus on the contributions AI can make to clinical health care, a domain that poses new and sometimes unique challenges to the application of AI. In the next sections, we discuss some important challenges and provide our recommendations on how to deal with them.

While radiology imaging was first in delivering digital data, digital pathology is a more recent revolutionary development [4,12,13]. In addition, for many years, hospitals have been digitizing their medical patient records [14]. Hence, a large and ever-increasing body of reasonably annotated clinical data has been collected: partially structured data in machine-readable formats, such as those from medical imaging, and partially unstructured data in natural language. As in other industrial sectors, it is expected that this *big data* movement can be leveraged to transform health care and drive unprecedented improvements in quality of patient diagnostics, treatment, care, and clinical outcome. Expected results range from identification of individuals at high risk for a disease, to improved diagnosis and matching of effective personalized treatment to the individual patient, as well as out-of-hospital monitoring of therapy response [15]. Although these opportunities and this potential are widely acknowledged, it is important to understand what can be delivered in practice with the current state-of-the-art AI technologies and which applications require further advances in AI to become feasible.

## Artificial Intelligence Methods: Supervised or Unsupervised Learning; Knowledge-Based or Data-Driven?

Multiple AI technologies are available to choose from [2,9,16]. Algorithmic learning-based AI can be performed in a supervised mode; this means that a *ground truth* label is available for every data sample, which guides the AI effort and is based on domain knowledge. It will be obvious that the correctness of *ground truth* labels is a prerequisite for good performance of an AI solution. The alternative, unsupervised mode, is when no *ground truth* is available and only similarities can be found with a yet undefined meaning [2,16].

Machine learning traditionally involves a human to determine features of the data, using domain knowledge. In contrast, deep learning allows finding such features from the data *by itself*. The features are subsequently used in various models. Some of those can be knowledge-based models in which new deep learning-defined features are integrated according to knowledge [17,18]. The current interest in AI from industry comes from the recent breakthroughs in data-driven approaches, such as deep learning, and their applicability in industrial applications such as speech recognition, machine translation, and computer vision. Still, it is expected that combining data-driven and knowledge-based approaches will bring AI to the next level, much closer to human intelligence [17,18].

## Artificial Intelligence for Well-Defined Narrow Tasks: Radiology Imaging and Digital Pathology

One of the more studied and successfully executed AI opportunities is in imaging. For example, AI technologies can be applied to problems such as distinguishing cell nuclei or certain cell types present in a tumor sample on a histopathology slide, using slide images obtained with a digital pathology scanner [19-21]. Such images are made with consistent equipment and acquired in a controlled fashion, generating images consisting of uniform data and providing very good representations of the phenomena to be modelled. The problem domain is limited. For instance, in the training process, the AI system gets as input raw images with associated labels for different cell types that are provided by a pathologist. The pathologist is providing a *ground truth* , based on existing expert knowledge (eg, on the different cell types or architecture present in the tissue slide). Deep learning has been applied to this problem and AI technologies already outperform manually crafted tissue analysis technologies [16,20,22,23]. It is expected that they will soon be on par or better than a human pathologist on certain well-defined histology feature recognition and measurement tasks, though not yet for clinical interpretation.

## Artificial Intelligence for Wider Purposes: Coupling Patient Clinical Data to Clinical Outcome

On the other hand, research projects are ongoing using multimodal data (ie, a combination of datasets of a different data type), for example, to enable prediction of prognosis of a patient or clinical outcome after a certain treatment. One may, for example, use medical imaging data combined with histopathology and clinical laboratory data and even lifestyle data to try to predict survival, risk of rehospitalization within a certain number of days, etc. Such projects remain challenging and have typically been proven not to be very successful [5,24]. IBM’s Watson for Oncology claims to integrate all available cancer patient data and disease information for improved diagnosis and therapy decision making. In 2013, the MD Anderson Cancer Center started using IBM Watson technology to increase effective treatment of cancer patients; however, the project was stopped in 2017 because it “did not meet its goals” [25,26]. In contrast, the concordance with respect to clinical interpretation of single-modality genome sequencing data using Watson for Genomics versus a clinical genomics expert group was reportedly quite good, between 77% and 97%, depending on the type of identified genomic mutations [7].

The most difficult challenge for AI in the coming years will be to move from successful narrow domains into wider-purpose, multimodality, data systems. A promising approach here is not to find one methodology to address every problem but to separate the wider-purpose AI goal into smaller goals. In this approach, subgroups of the data may be processed separately with suitable AI methods to provide meaningful, clinically relevant output. For example, for cardiac ultrasound images, one could zoom in to develop an algorithm with deep learning to measure left ventricular volume; or for pathology slide images, one could zoom in to develop an algorithm for recognition and quantification of a specific cell type (eg, lymphocytes). To increase chances at success, it is important to determine a well-defined, focused, and clinically relevant question for an AI project that can be adequately answered with the available data.

The conclusion of this section is that a relevant and well-defined clinical question should come first.

## The Relationship Between Patient or Sample Numbers and Data Variables

Many AI techniques, especially deep learning, rely on the availability of large datasets or *big data* [15]. Sometimes domain knowledge can help to create additional data derived from the data that are available. It is important, however, to distinguish the type of data that is needed. In games, such as chess and Go, it is easy to artificially synthesize additional data of the right type to increase the size of the dataset. With respect to medical and histopathology imaging, large amounts of data are available since samples are defined on an image pixel basis. Using this type of data, with relatively few images one can create millions of annotated samples with drawing tools. It is relatively easy to further augment every sample with artificially generated variations (eg, mirror copies, rotated versions, modified intensities, and modified colors) without consequences for the annotation.

In contrast, in clinical health care the type of data typically is a pathology or radiology report from a patient, associated with a clinical annotation such as diagnosis or response to therapy. In this case, the number of samples is generally equal to the number of patients. The annotation is often more difficult, as it requires an expert physician to provide the *ground truth* . When using multimodal data to find parameters that, for example, predict clinical outcome, despite all digital records and digital health devices, there are not enough data. The number of patients for which the necessary multimodal data are available is, in general, the limiting factor for using AI methods on such combined data sources to create a valid algorithm for risk prediction, diagnosis, or a therapeutic decision. When the number of patients of a specific defined disease (sub)type is low, the often-heard strategy is to extend a study to include more patients, even all patients worldwide, which requires addressing various legal and technical barriers. However, this is still likely to fail in reaching the required patient number; with efforts to increase the number of patients for inclusion in data analysis, the amount of variation per patient, including many unknown features and variables, tends to grow as well, leading to uncontrolled data variation. This is caused by the large variation in human individuals: their DNA (ie, just think of the 3 billion base pairs and the near-infinite combinations of genomic variations), their lifestyle, family medical history, use of medication, etc. Moreover, patients are never treated in exactly the same manner in the various hospitals, bringing in many additional variables. It is a well-recognized issue in clinical trials run by pharma companies [24]. The challenge is to minimize such unwanted variables in the patient or sample set to analyze. Much of this uncontrolled variation is not recorded or, at best, only in a very noisy way. The number of unknown parameters that may have influenced the outcome, especially if its measurement lies many years after the diagnosis and treatment, is typically underestimated. Examples of failure of AI methods caused by these issues include many genome-wide association studies aimed at identification of clinically useful genomic risk factors for complex diseases and genomic studies aimed at identification of biomarkers for cancer diagnostics and treatment decisions [27].

Similar challenges are present in other domains, but solutions in those areas can be invoked that are not possible in the health care domain. In natural language processing, Google Translate is a well-known example. When it started, translations were of very poor quality and heavily criticized, but Google decided to keep the service up and running; online feedback was used to collect a large amount of translation data, enabling continuous improvement of the performance of the translation algorithm [28].

In summary, for applying AI to multimodal patient data, the number of patients from whom the complete set of multimodal data is available is frequently too limited to address the *curse of dimensionality*. In the scientific community, dimensionality reduction remains an active research area [29-31]. In clinical application areas for AI, it remains the main challenge to address. The first solution lies in reducing data modality and bringing the number of variables (*P*) on the right level in relation to the number of patients or samples (*N*) for which a *ground truth* is available. The desired solution will reduce high-dimensional data to biologically sound knowledge-based features. Introducing knowledge-based computational approaches is expected to provide a way forward to reduce model freedom and handle high-dimensional data [32].

The conclusion of this section is that the ratio between the number of patients and their variables should fit the AI method.

## Insufficient Data Quality and Many Subjective Parameters

For the patient data that is available, it turns out that this data is usually neither 100% complete nor 100% correct. For example, diagnoses are not always complete or correct, or they were not correctly entered into the digital domain. The main diagnosis is, in general, reasonably well-documented; however, side diagnoses and complications that arise, for example, during hospital admission or in the home setting, as well as treatment details are less-accurately or not documented [5]. For many clinical variables, such as diagnoses, the *ground truth* comes from a physician’s judgement and cannot be objectively measured or quantified. For example, it is documented that histopathology diagnoses differ to a varying extent among pathologists that diagnose the same slide [33-35]. As a consequence, datasets may be incomplete and noisy, and presumed *ground truths* may not always be correct.

The conclusion of this section is that the right data (ie, representative and of good quality) needs to be obtained.

## Causal Relations Versus Correlations: A Role for Bayesian Reasoning

Any data-driven approach on data for which the ratio of number of patients (ie, samples) to variables is too low can lead to multiple spurious correlations [36]. This means that the data suggest a correlation between two factors, but this is purely due to chance and there is no underlying explanation or causal relationship. In machine learning, this can easily lead to overfitting and finding of irrelevant correlations [16,37]. Also, for clinical implementation, any interesting correlation (eg, a feature or combination of features associated with increased disease risk) needs to be clinically validated at high cost, where lack of causality generally results in very low success rates. Therefore, turning an algorithm, based on correlations, into a successful proposition will, in general, be easier if causal relations underlie the found correlations. Knowledge-based reasoning techniques, such as Bayesian network models, can reduce the number of spurious relations and overfitting problems by using existing knowledge on causal data relations to eliminate noisy data [11,14]. As an additional advantage, Bayesian models can deal very well with uncertainty and missing variables, which is the rule rather than the exception in clinical data [11]. Not a coincidence, with respect to patient data interpretation, Bayesian models reason the way a medical doctor does [38].

The conclusion of the section is that the relationship between data and *ground truth* should be as direct and causal as possible.

## Validation of Artificial Intelligence-Based Solutions

For many of the success stories of AI, a robust and reliable result is usually not necessary. For a free translation service, the consequence of a wrong decision is at most a dissatisfied customer. Improvements of those services could happen relatively quickly because many of those AI applications are deployed in the field and iteratively improve their performance on the basis of new data, thus learning from their mistakes. In sharp contrast to most of these consumer or lifestyle solutions based on AI, every clinical application, be it hardware or software, requires a thorough clinical validation in order to be adopted by the professional clinical community for use in patient care, such as diagnostics or treatment decisions, and must be approved by regulatory authorities [24]. The requirements for clinical validation will be more stringent when errors or mistakes can have greater consequences. In a clinical trial, it needs to be demonstrated how accurately the developed AI solution performs compared to the clinical standard (eg, sensitivity and specificity of a diagnostic test). Still, it is not completely clear whether good performance of an algorithm is acceptable if the solution is a “black box” and not transparent and rationally explainable [2]. On top of that, it is not obvious what proper validation of a continuous learning-based solution implies. An important issue is that because of lack of transparency, deep learning-based “black box” algorithms cannot be easily improved, in contrast to, for example, Bayesian models that are based on a transparent structure. Initial attempts to tackle this challenge are on the way [39].

Have AI-based solutions already been approved for clinical use? The earlier mentioned Watson for Oncology system operates as a “black box” and its advice could not be clinically validated [26]. On the other hand, in 2017 it was claimed that the first deep learning-based algorithm, which identifies contours of cardiac ventricles from a magnetic resonance imaging (MRI) image to calculate ventricular volume, was validated and approved by the US Food and Drug Administration (FDA) for performing the calculation faster than a clinician [40]. Obviously, this system’s scope is far more restricted than Watson’s; the unimodal imaging data that were used were directly and causally related to the *ground truth* provided during every image analysis by the clinician. Also, it can be considered a measurement algorithm and does not include a clinical interpretation claim. Clinical validation and obtaining regulatory approval are much more difficult for those algorithms for which such an interpretation claim is added [4].

Several new solutions are ready or able to perform continuous (ie, incremental) learning [41]. However, within current regulations, an AI system for clinical applications should be “frozen” and can, therefore, not learn online and immediately apply its new knowledge. Rather, it needs to have an offline validation of the obtained “frozen” model on an independent series of patient or sample data. Following a next continuous-learning cycle, the validation process needs to be repeated again prior to renewed implementation of the model. Ideally, new clinically acceptable ways to shorten validation tracks for digital applications in a patient-safe manner should be found; it is expected that special procedures will be put in place to facilitate regulatory approval of updated algorithms. In line with this, the FDA is actively developing a strategy to deal with AI-based software solutions [42]. Maximal use of existing knowledge in transparent and causal model algorithms, as in Bayesian modeling, is expected to facilitate both clinical validation and obtaining regulatory approval, both for unimodal as well as for multimodal data.

The conclusion of this section is that procedures must be put in place to facilitate algorithms to be regulatory ready and to enable validation.

## Methods to Use

Technology-wise, numerous methods from the domain of AI have been explored for the development of clinical applications [8,11,16,43]. Some have been more successful than others, mostly depending on application type. For automating pathology diagnosis using tissue slide images, deep learning has proven to be an appropriate technology. When dealing with more general multimodal problems, such as predicting clinical outcomes, patient assessments, and risk predictions, other methods that often include domain knowledge are likely to be more appropriate choices. Probabilistic methods using knowledge representation are increasingly used and enable reduction of the number of influencing variables, determining *sensible* features or latent variables. Probabilistic Bayesian modeling is well-suited to deal with complex biological (eg, “omics” data, such as genomics and transcriptomics data) as well as medical and clinical data; it is finding its way into diagnostic applications as well as drug development [9,44-49]. However, where knowledge is lacking, knowledge-agnostic AI approaches become valuable; Bayesian reasoning networks are thought to have high potential for use in combination with deep learning, combining the best of two worlds in Bayesian deep learning [17,18,50].

The conclusion of this section is that the right AI method must be used for the problem.

## Recommendations for Use of Artificial Intelligence Methods to Develop Clinical Applications: The Six Rs

In view of the challenges related to the use of AI for health care and biomedical applications, we believe it will be of value to have some guidelines when designing a study. They may also serve to facilitate communication between scientists involved in AI and medical doctors. From the discussion above, we have extracted six basic recommendations.

1. Relevant and well-defined clinical question first. Data analytics without domain knowledge can be applied in the health care domain, but at high risk of getting clinically irrelevant outcomes. For every new AI project, the clinical questions should be well-defined and reviewed with clinical experts. The outcome of the analysis should also be reviewed for clinical and/or biological sense.
2. Right data (ie, representative and of good quality). Carefully define the dataset that is needed to answer the clinical question. A clinical dataset with *ground truth* should be sufficiently clean and reliable. Be aware of hidden variation between samples that is not visible in the dataset. The dataset should be appropriate for the question at hand as well as representative for the population under study.
3. Ratio between number of patients and their variables should fit the AI method. To obtain useful results, ensure working with adequately large datasets (ie, numbers of patients or samples) for the AI method to be used, and reduce patient variables where possible. Use domain knowledge to limit spurious correlations.
4. Relationship between input variables and predicted output variable, as the dependent value, should be as direct and causal as possible. The clinical question should as closely as possible relate the *ground truth* to the data. Hence, finding new pathology features that best distinguish between two different pathology diagnoses can be successful; using lifestyle information to predict 10-year survival might not.
5. Regulatory ready; enabling validation. Upfront, consider how a certain solution can be validated and pass regulatory requirements. Consider how using domain knowledge could speed up the validation process, for instance, by breaking up the AI system into smaller AI systems. This effectively excludes systems that iteratively change by continuous learning.
6. Right AI method. Use the right method for the question at hand. Data-driven methods can be used if the data available allows it, and knowledge-based methods can be applied if there is knowledge available but not enough data; a mixture of the two, combined in a wise manner, may be highly productive for development of clinically applicable health care solutions.

## Privacy Issues

Driven by the *big data* analysis developments in health care, new privacy regulations were recently implemented in Europe—General Data Protection and Regulation (GDPR) [51]. To protect privacy, individuals control their own personal data, and explicit informed consent is required for access to the data and use in AI. This regulation is expected to make it more difficult to share patient data between multiple medical centers and with companies involved in development of AI solutions.

## Key Takeaways

While AI approaches are excellently suited to develop algorithms for analysis of unimodal imaging data (eg, radiological or digital pathology images), for clinical (ie, patient-related) applications, major challenges lie in the usually limited patient or sample numbers (*N*). This is in comparison to the number of multimodal variables (*P*) due to patient variation, inadequate *ground truth* information, and a requirement for robust clinical validation prior to clinical implementation. Artificial Intelligence solutions that combine domain knowledge with data-driven approaches are, therefore, preferable over solutions that use only domain knowledge or are fully data driven. We introduce the following 6R model to keep in mind for AI projects in the biomedical and clinical health care domain:

1. Relevant and well-defined clinical question first.
2. Right data (ie, representative and of good quality).
3. Ratio between number of patients and their variables should fit the AI method.
4. Relationship between data and *ground truth* should be as direct and causal as possible.
5. Regulatory ready; enabling validation.
6. Right AI method.

## Abbreviations

AI: artificial intelligence
FDA: US Food and Drug Administration
GDPR: General Data Protection and Regulation
MRI: magnetic resonance imaging
